# Supplementary material for: DMRforPairs: identifying Differentially Methylated Regions between unique samples using array based methylation profiles
Source: BMC Bioinformatics. 2014 May 15;15:141. doi: 10.1186/1471-2105-15-141 (PMC4046028; doi:10.1186/1471-2105-15-141)
Supplement: Additional file 2 — DMRforPairs output for the comparison of A431-MCF7 and NA17018-NA17105. Please start from the HTML files in each folder. Available via the BMC Bioinformatics website. [file 1471-2105-15-141-S2.zip › 1394847754114233_MOESM2_ESM/A431_MCF7/figures/5634.pdf]

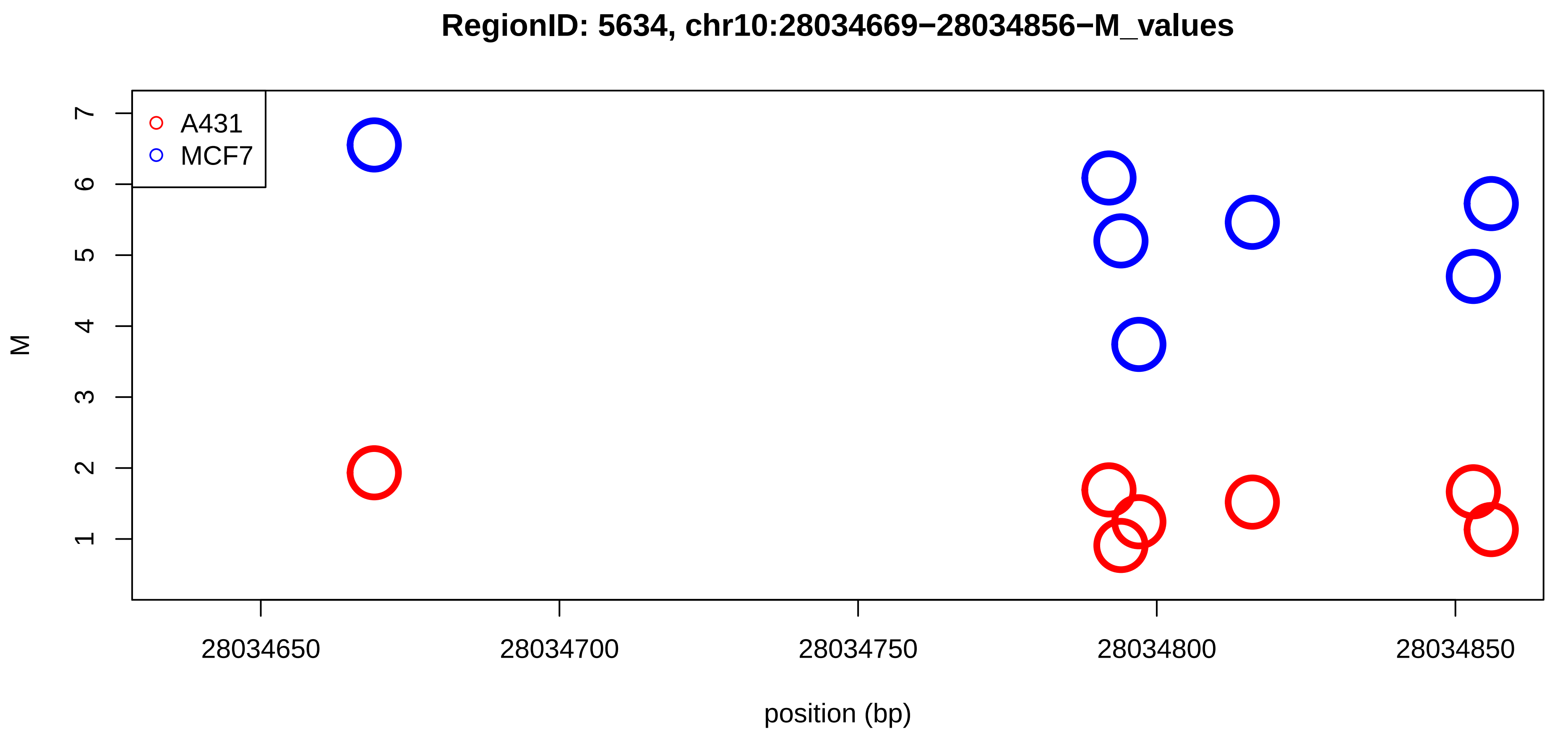
RegionID: 5634, chr10:28034669−28034856−M_values
A431
MCF7
M
position (bp)
28034650
28034700
28034750
28034800
28034850

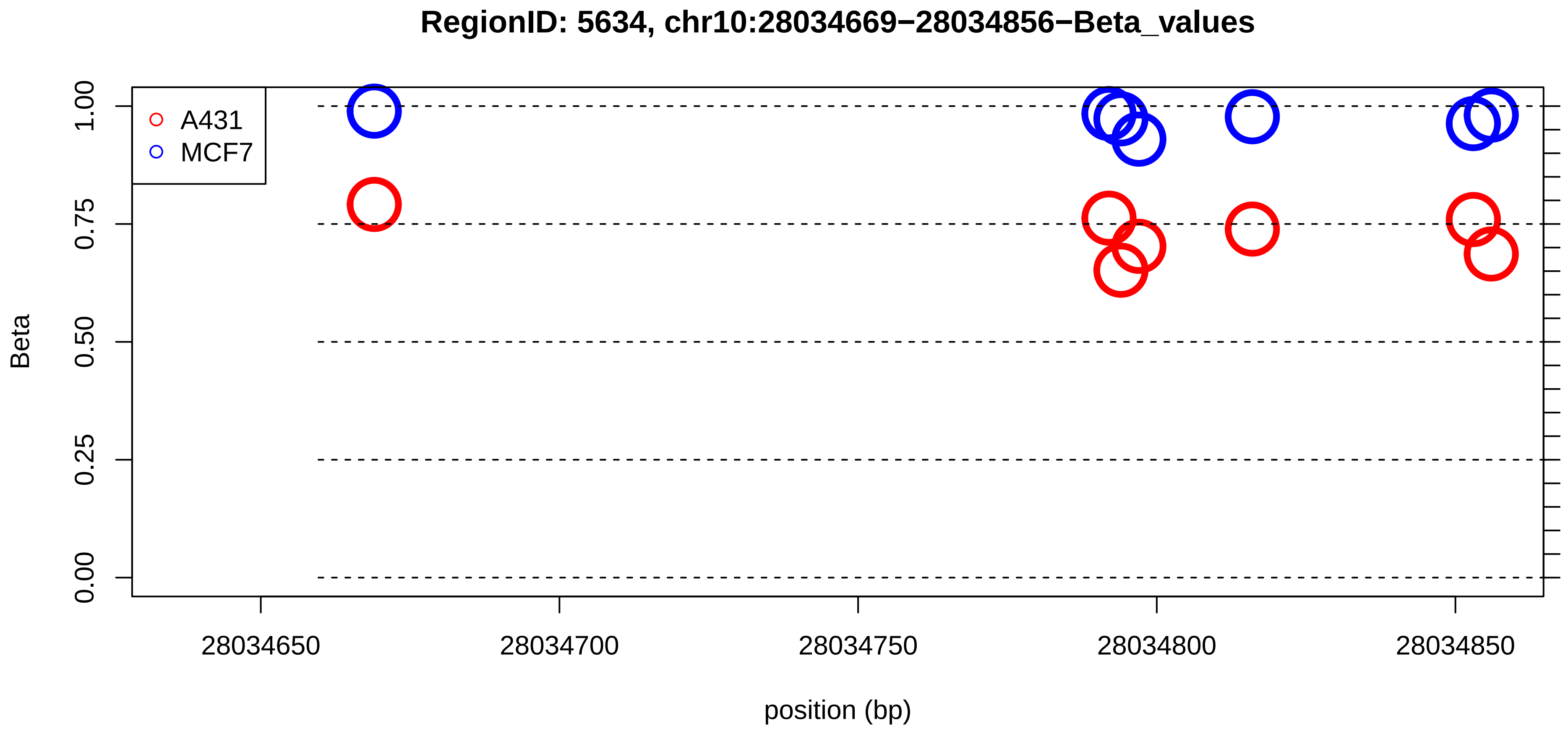
RegionID: 5634, chr10:28034669−28034856−Beta_values
A431
MCF7
Beta
position (bp)
28034650
28034700
28034750
28034800
28034850

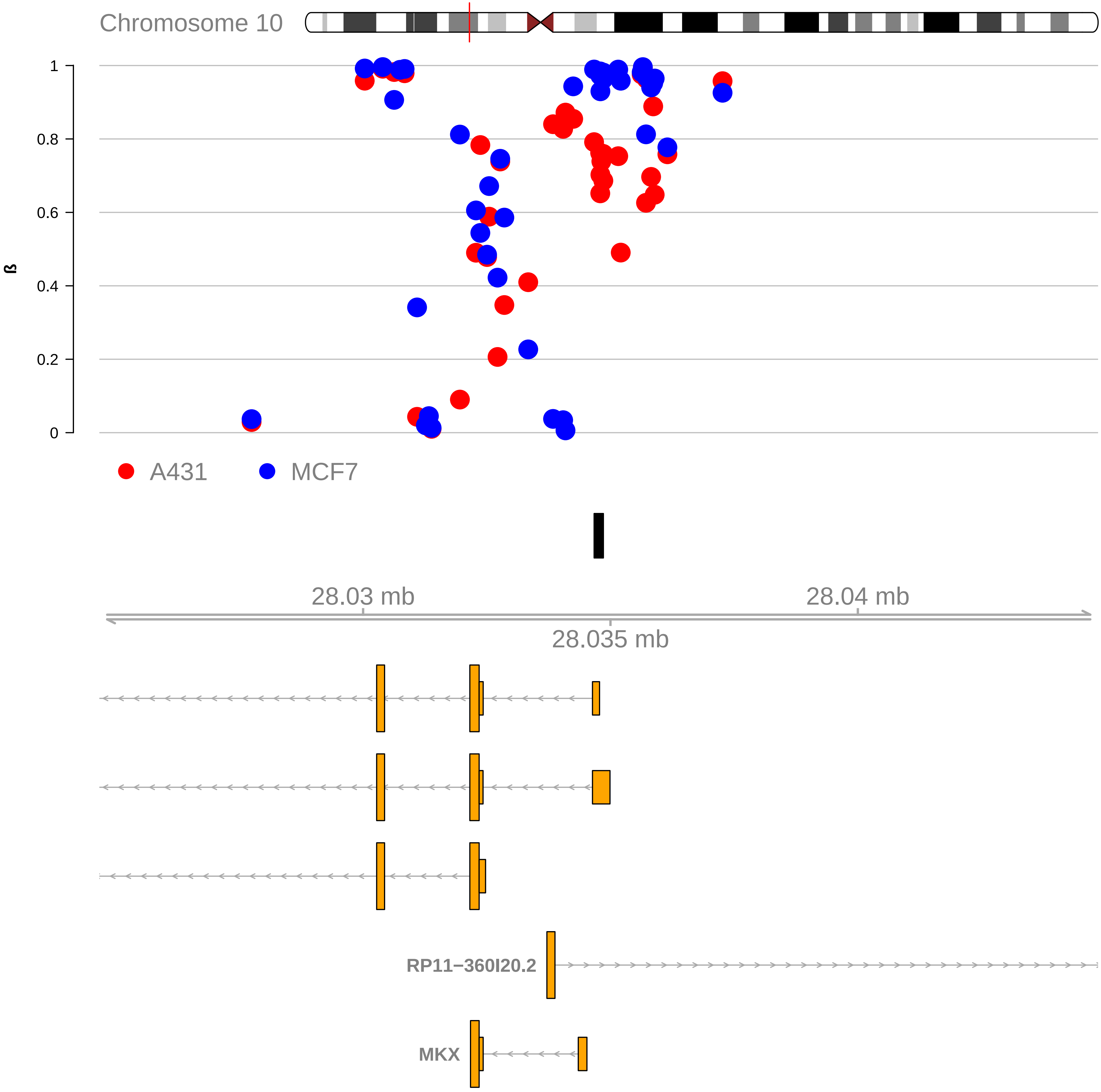
Chromosome 10
ß
1
0.8
0.6
0.4
0.2
0
A431
MCF7
28.03 mb
28.04 mb
28.035 mb
RP11−360I20.2
MKX
